# A Fast and Inexpensive Protocol for Empirical Verification of Neutralizing Epitopes in Microbial Toxins and Enzymes

**DOI:** 10.3389/fvets.2017.00091

**Published:** 2017-06-13

**Authors:** Christine N. Vuong, Wen-Ko Chou, Vivek A. Kuttappan, Billy M. Hargis, Lisa R. Bielke, Luc R. Berghman

**Affiliations:** ^1^Department of Veterinary Pathobiology, Texas A&M University, College Station, TX, United States; ^2^Department of Poultry Science, Texas A&M University, College Station, TX, United States; ^3^Department of Poultry Science, University of Arkansas, Fayetteville, AR, United States; ^4^Department of Animal Science, The Ohio State University, Columbus, OH, United States

**Keywords:** epitope mapping, antibody production, *Clostridium perfringens*, alpha-toxin, antibody-guided, CD40, poultry

## Abstract

*In vivo* targeting of peptides to antigen-presenting cells by use of agonistic anti-CD40 monoclonal antibodies has been used successfully as an immune response enhancing strategy. When tested in chickens, the antibody-guided platform was capable of inducing specific IgG production within 1 week postimmunization. However, use of this method beyond its initial conception as a vaccine delivery tool has not been fully exploited. In this study, *Clostridium perfringens* alpha-toxin was used as a model microbial toxin for epitope mapping by using the antibody-guided immunization method to generate a panel of antibodies against specific, regions of the toxin in an attempt to identify crucial determinants on the toxin which, once bound, would hinder downstream toxicity. Alpha-toxin, which possesses both hemolytic and phospholipase C (PLC) enzymatic activities, has long been known to be one of the key destructive etiological agents of necrotic enteritis disease in poultry. Previous attempts to identify crucial antigenic determinants on the toxin mediating its enzymatic activities have been performed using expensive and labor-intensive site-directed mutagenesis techniques. To create a panel of antibodies, 23 short candidate alpha-toxin peptide regions were selected *in silico* using B-cell epitope prediction algorithms in the public domain and were custom synthesized to load onto the antibody-guided complex for immunization in birds for antisera production. Peptide-specific antibody responses were generated against all candidate neutralizing epitopes and used for *in vitro* toxin neutralization tests. Antisera against all 23 peptides were able to neutralize the toxin’s hemolytic activity, with neutralization titers ranging from 80 to 320, but none were effective in blocking PLC. The novel approach of antibody-guided immunization introduces a new, inexpensive method for polyclonal IgG production and *de facto* identification of neutralizing epitopes in microbial toxins and enzymes within 2 weeks from *in silico* analysis of a putative target sequence.

## Introduction

Inducing antigen-presenting cell (APC) activation and effector responses require both binding of a specific antigen by the APC and co-stimulatory signals received from helper T-cells. Binding and activation of the CD40 receptor on B-cells emulate the germinal center environment and trigger downstream rapid antibody production and isotype switching against a specific antigen (1). Manipulation of this system would be beneficial for controlled guidance of the immune system’s reactivity against a defined target, specifically by using agonistic monoclonal antibodies against the CD40 receptor loaded with an antigen of interest. These CD40-targeting antibody-guided complexes have previously been tested for use as *in vivo* delivery systems for vaccines (2–4). In poultry, this CD40-targeting approach has been shown to induce robust and specific IgG serum antibody responses within 1 week (5), as well as sIgA production in the mucosal samples (6), essentially bypassing the weaker, chiefly IgM, initial immune response associated with primary immunizations. The application of this guided complex to induce rapid antibody production beyond its initial vaccine designation has not been exploited. To assess the capabilities of the antibody-guided immunization system, *Clostridium perfringens* alpha-toxin (Cpa) was used as a model microbial toxin for rapid antiserum production and downstream epitope mapping.

Alpha-toxin is one of many toxins produced by Clostridial bacteria and possesses both hemolytic and phospholipase C (PLC) enzymatic activities, making it an ideal model for epitope mapping. Neutralizing antibodies can be produced against specific regions of the toxin to test the antibody’s ability to inhibit one or both of the toxin’s enzymatic functions. In poultry, *C. perfringens* is the causative agent responsible for necrotic enteritis and continues to be an obstacle for the industry (7, 8). Although part of the commensal gut flora, *C. perfringens* can cause disease when an altered gut microenvironment or pre-established intestinal damage facilitates abnormal overgrowth and microbial dysbiosis in the gut (9). This imbalance results in intestinal lesions caused by the bacterium’s multiple toxins and leaky gut syndrome in the bird (10, 11). Although alpha-toxin is no longer considered the sole toxin to target for vaccine development (12), a rapid method to determine the regions required to neutralize a toxin’s activities would be of significant interest. Previous epitope mapping studies have primarily utilized site-directed mutagenesis, but this method requires specific base changes, molecular cloning, and downstream expression and purification before the altered toxin can be tested for change in function (13–16). Introduction of a less expensive and more rapid epitope mapping method would be beneficial for researchers attempting to identify essential regions on a protein or candidate targets for therapeutics.

In this study, Cpa was used as a model microbial toxin for epitope mapping to determine whether the antibody-guided immunization method has potential to be used for rapid identification of targets for downstream toxin neutralization or vaccine development. A panel of linear peptide epitopes spanning the majority of the Cpa’s amino acid sequence was synthesized. The synthetic peptides were incorporated into the antibody-guided immunogen complex and administered in chickens for polyclonal IgG production. The peptide-specific antisera produced were used for downstream *in vitro* neutralization testing against the toxin’s hemolytic and PLC enzymatic functions, respectively. Using Cpa as a model toxin, this approach expands the function of antibody-guided immunization complexes beyond its initial use as a delivery system in poultry and highlights its potential as a method for rapid IgG production/reagent development and as the fastest method to deliver proof of concept of potential toxin and enzyme neutralization strategies.

## Materials and Methods

### Peptide Epitope Design

**Figure 1:**
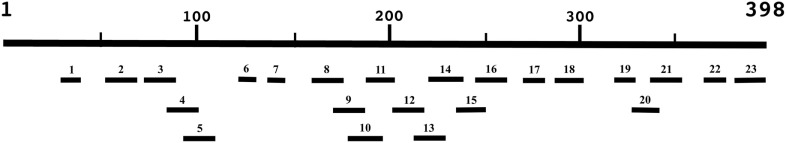
Schematic of 23 peptides generated based on the *Clostridium perfringens* alpha-toxin amino acid sequence (CAA35186.1). Linear peptides were selected based on the ease of synthesis using Immune Epitope Database and Analysis Resource publically available B-cell epitope prediction algorithms (figure not to scale) (17).

Hydrophilic segments ranging from 9 to 23 amino acids in length were designed based on the 398 amino acid Cpa sequence (GenBank Accession CAA35186.1) using Immune Epitope Database and Analysis Resource open-source predictive algorithms to construct a peptide library (17). The library consisted of 23 peptide epitopes in order to provide maximum coverage of the primary structure of the toxin while maintaining ease of synthesis (Table 1); peptides were designated as numbers 1–23 based on starting position on the original Cpa toxin sequence (Figure 1). Hydrophobic stretches of the Cpa toxin were omitted to avoid peptide synthesis issues. Only consecutive linear regions of the alpha-toxin were selected for synthesis to avoid the time and expense associated with the protein expression and purification required to produce conformational epitopes. Biotinylated commercially synthesized peptides (Genscript, Piscataway, NJ, USA) were incorporated stoichiometrically in the antibody-guided immunization complex as described previously (5).

**Table 1:** *Clostridium perfringens* alpha-toxin-derived synthetic peptides.

Peptide #	*Clostridium perfringens* alpha-toxin (Cpa) toxin start position	Length	Sequence
1	31	9	GKIDGTGTH
2	51	15	ENDLSKNEPESVRKN
3	71	20	ENMHELQLGSTYPDYDKNAY
4	81	20	TYPDYDKNAYDLYQDHFWDP
5	91	20	DLYQDHFWDPDTDNNFSKDN
6	117	10	IPDTGESQIR
7	136	10	EWQRGNYKQA
8	158	23	DIDTPYHPANVTAVDSAGHVKFE
9	170	20	VDSAGHVKFETFAEERKEQY
10	181	20	TFAEERKEQYKINTAGCKTN
11	191	21	KINTVGCKTNEDFYADILKNK
12	200	20	EDFYADILKNKDFNAWSKEY
13	210	20	KDFNAWSKEYARGFAKTGKS
14	220	17	ARGFAKTGKSIYYSHAS
15	233	17	SHASMSHSWDDWDYAAK
16	240	20	SWDDWDYAAKVTLANSQKGT
17	270	16	DVSEGNDPSVGNNVKE
18	291	12	STSGEKDAGTDD
19	309	13	KTKDGKTQEWEMD
20	320	21	DNPGNDFMAGSKDTYTFKLKD
21	330	20	SKDTYTFKLKDENLKIDDIQ
22	354	16	RKRKYTAFPDAYKPEN
23	379	19	VVDKDINEWISGNSTYNIK

*Design based on Cpa GenBank Accession: CAA35186.1 amino acid sequence*.

### Antibody-Guided Immunogen Complex

Immunization complexes were produced as previously described by Chen et al. (5). Biotinylated CD40-targeting antibodies were complexed with each synthetic peptide using streptavidin as a scaffold. Antibody-guided complexes were stoichiometrically produced to contain a molar ratio of 2 antibodies and 2 peptides to a single streptavidin. Non-targeting complexes were also produced by replacing CD40-targeting antibody with normal (non-targeting) mouse IgG and served as negative controls. Non-targeting control complexes incorporated either peptide #13 or #14 and were further designated as 13C and 14C, respectively.

### Immunizations

A total of 75 6-week-old broilers were divided into sets of three, creating a total of 25 groups. Animal care and handling was approved by Texas A&M University Institutional Animal Care and Use Committee (permit #2013-0254). Because chickens are outbred animals and were expected to exhibit divergent immune response levels, each peptide candidate was administered to three birds to ensure at least one good responder. Pre-immune serum was collected from all birds and designated as Day 1 samples. Each group of birds was subcutaneously immunized with 50 μg of antibody-guided complex carrying 1 of the 23 peptides. Two extra groups of birds were immunized with non-targeting antibody complex (as negative controls), using either peptide #13 or #14, and are further referenced as control groups 13C or 14C. Serum was collected 1 week postimmunization and designated as Day 7 samples; these samples were used for *in vitro* antibody titer measurements and toxin neutralization assays.

### ELISA

Goat anti-biotin IgG and the biotinylated target peptides were pre-mixed at a 1:1 M ratio. This pre-mix was coated onto 96-well microtiter plates at a concentration of 5 μg/mL in carbonate-bicarbonate coating buffer, pH 9.6. Wells were blocked with 5% (w/v) BSA in PBS and serum samples were applied at 1:50 dilution. Samples were incubated for 2 h at 37°C before peroxidase-conjugated goat anti-mouse IgG secondary antibody was applied (Jackson ImmunoResearch, West Grove, PA, USA). Peroxide/tetramethylbenzidine substrate system was used as the colorimetric endpoint and enzymatic reactions halted with 2 M sulfuric acid. Absorbances were read at 450 nm using a Perkin-Elmer Victor 2 plate reader (Waltham, MA, USA). Antibody titers were reported as Day 7:Day 1 ratio to correct for interference from pre-existing cross-reactive antibodies in circulation. No statistical analysis was performed as only qualitative responses, production of any neutralizing antibodies for use in downstream assays, were needed for the study.

### Hemolytic Neutralization and PLC Neutralization Assays

Purified Cpa was obtained from USDA APHIS and used at a working dilution of toxin in sterile PBS for neutralization assays, as recommended by the manufacturer. Antisera from the two highest responders of each group, based on previously performed ELISA, were used for hemolytic neutralization testing. Sera were titrated by 2-fold serial dilution starting from an initial 1:10 starting dilution on a microtiter plate in a 50 µL volume, and then mixed 1:1 v/v with the working stock of alpha-toxin. Toxin and sera were incubated at 37°C for 1 h to allow potential binding/neutralization of the toxin. After initial incubation, 100 µL of 5% (v/v) sheep red blood cells diluted in PBS was added and incubated for another hour at 37°C. After incubation, neutralization of hemolytic activity was observed. PLC neutralization assays were performed using the same procedure, but modified for the application of 10% (v/v) egg yolk emulsion as a source of phospholipids, in lieu of red blood cells. Neutralization titers are reported as the inverse of highest serum dilution factor capable of fully neutralizing the enzyme. Because each peptide candidate was only represented by two antisera samples and reported in inverse dilution factor, SEs are not included. Statistics to compare between groups was not performed, as only qualitative data showing ability to neutralize were needed to determine whether a specific region is a suitable target or not; antibodies against any region able to neutralize both enzymatic activities would have been considered as an indicator for potential candidate after epitope mapping.

## Results

### Peptide-Specific Polyclonal IgG Rapidly Produced Using Antibody-Guided Immunization

**Figure 2:**
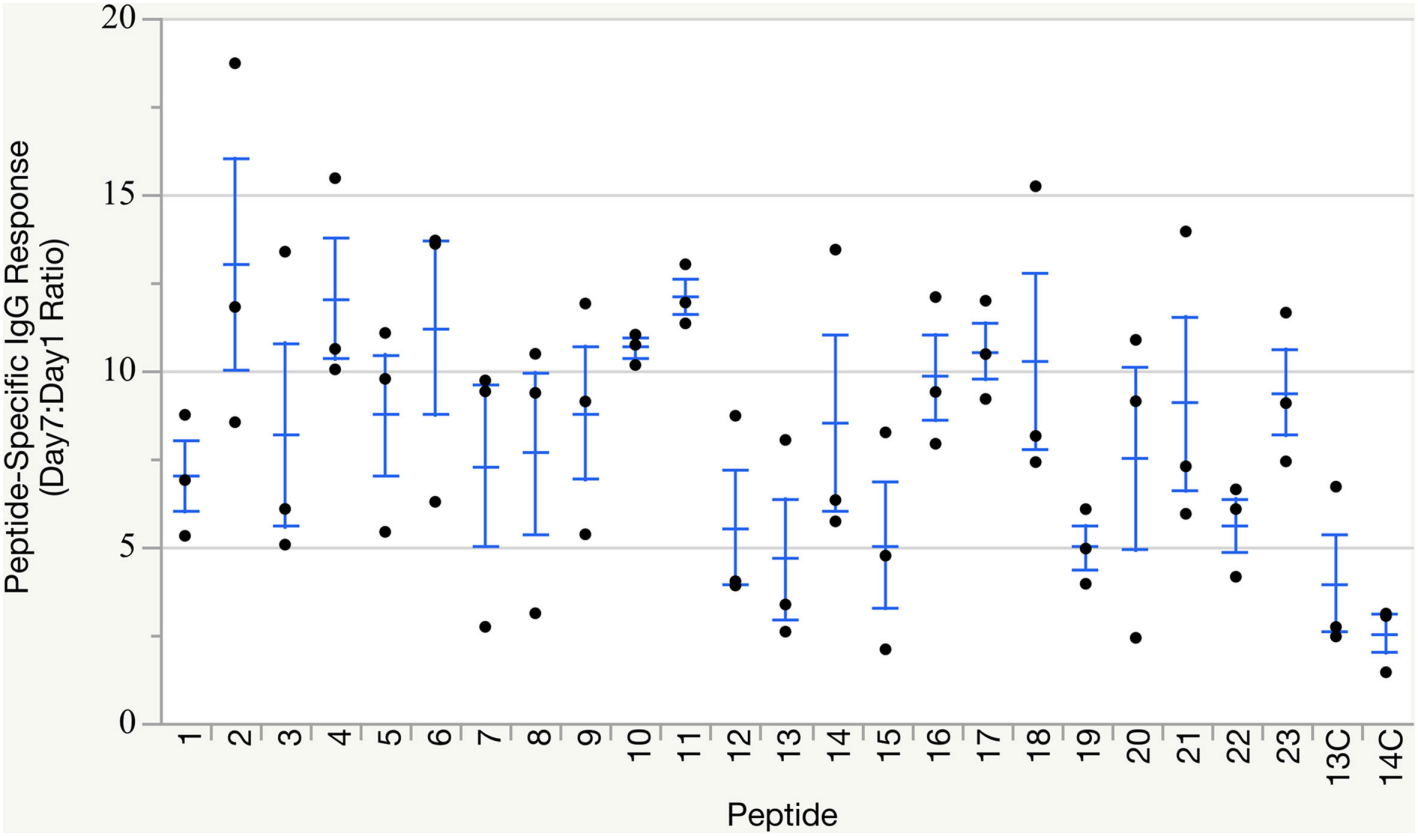
Peptide-specific IgG responses. Individual peptide-specific antibody titers reported as Day 7:Day 1 ratio based on ELISA measurements against the matching peptide used for immunization. Groups 13C and 14C were immunized with non-targeting complexes loaded with either peptide #13 or #14 and served as non-targeting negative controls. Group means and SE overlaid.

All groups of birds mounted humoral immune responses against their respective peptide immunogen within 7 days of immunization (Figure 2), as measured by peptide-specific IgG titers via ELISA. As expected, control groups receiving peptide loaded onto non-targeting complexes also mounted low-level antibody responses against the peptide, but these responses did not reach the overall robust levels induced by CD40-targeted peptide delivery. Individual immune responses varied, as anticipated from outbred birds. Statistical analysis to compare response between groups was not necessary as only qualitative responses, production of any neutralizing antibodies for use in downstream assays, were needed for this study.

### Blocking/Binding Cpa Linear Epitopes Sufficient for Neutralizing Hemolytic Activity, But Not PLC Activity

**Figure 3:**
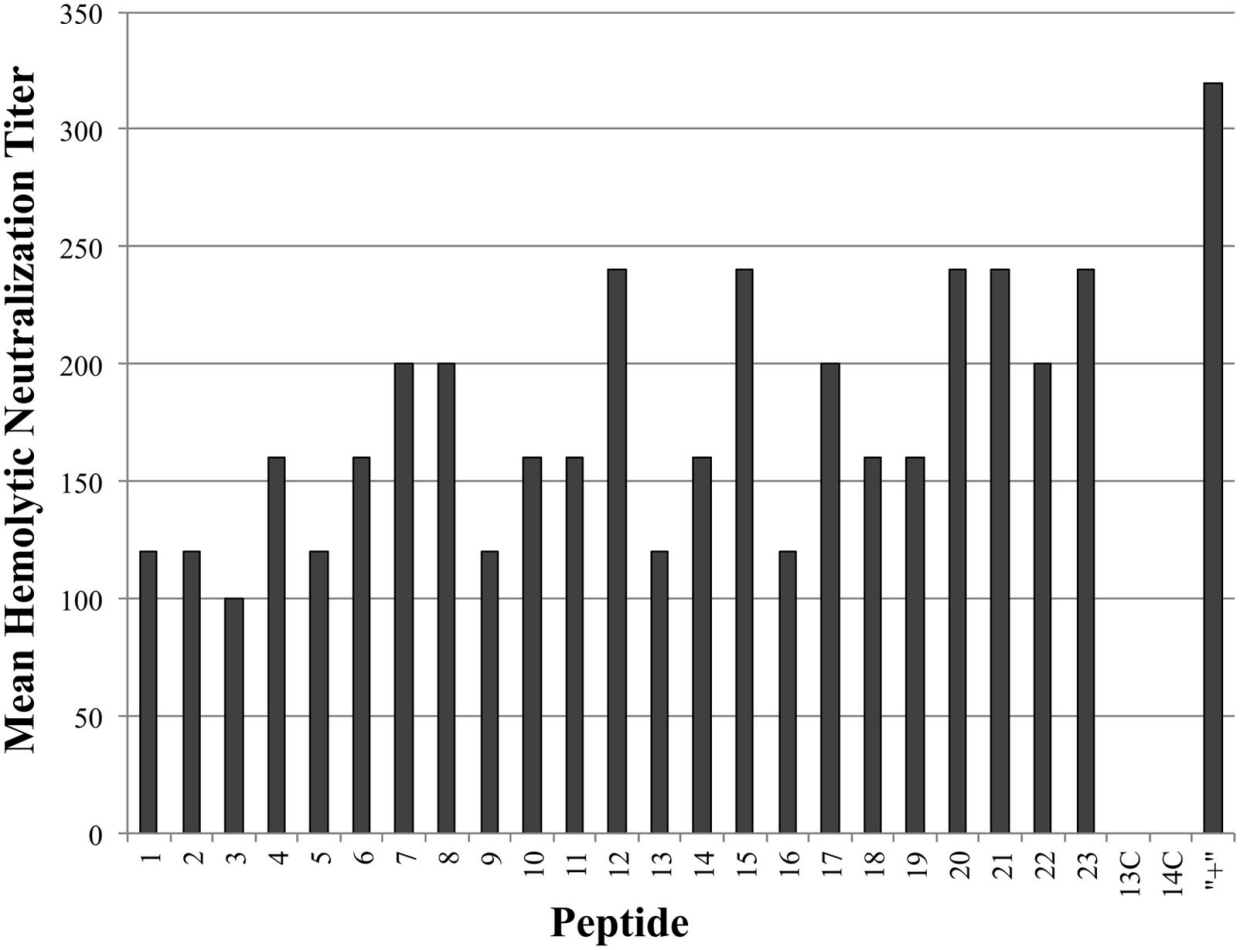
Mean hemolytic neutralization titers. Hemolytic neutralization titers reported as the inverse of the highest serum dilution factor capable of completely neutralizing the hemolytic activity of *Clostridium perfringens* alpha-toxin. Hyperimmune serum against alpha-toxin obtained from USDA APHIS was used as positive control serum and the corresponding group was labeled as “+” on chart.

**Figure 4:**
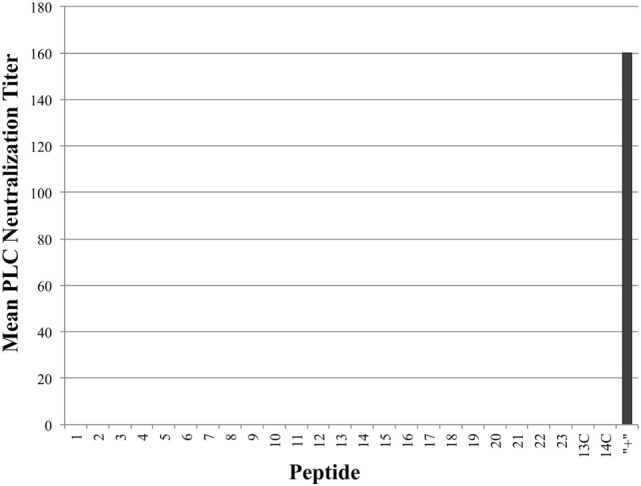
Mean phospholipase C (PLC) neutralization titers. PLC neutralization titers reported as the inverse of the highest serum dilution factor capable of completely neutralizing the PLC activity of *Clostridium perfringens* alpha-toxin. Hyperimmune serum against alpha-toxin obtained from USDA APHIS was used as positive control serum and the corresponding group was labeled as “+” on chart.

As seen in Figure 3, all tested antiserum samples were able to neutralize *in vitro* Cpa hemolytic activity to varying degrees (individual titers ranging from 80 to 320), suggesting that antibody binding of any accessible region on the toxin itself is sufficient to block hemolytic activity. Of note, groups 13C and 14C also produced some peptide-specific antibodies, as measured by ELISA, but these antibodies were unable to neutralize hemolytic activity. This suggests that antibodies produced against epitopes loaded onto antibody-guided complexes have gone through some affinity maturation and bind more efficiently than the immunization using the non-targeting counterparts. In contrast to the hemolytic neutralization results, none of the serum samples from the experimental groups were able to neutralize Cpa’s PLC activity (Figure 4). Hyperimmunized chicken antisera against native *C. perfringens* obtained from USDA APHIS were used as a positive control in neutralization assays and were capable of neutralizing PLC activity. The hyperimmune antisera would possess an assortment of antibodies against various regions and spatial conformations of Cpa, implying that the critical site responsible for PLC activity cannot be emulated by a synthetic linear peptide.

## Discussion

Antibody-guided complexes were initially designed for use as a vaccine delivery system, but this technique clearly also has potential to become an analytical tool to rapidly dissect molecules into their various active domains and pinpoint essential motifs underlying specific biological activities. This system has the added advantages of being both quicker and more cost-effective than standard site-directed mutagenesis procedures. The antibody-guided method used in this experiment was purposely limited to presenting linear epitopes; it appeared that these did not produce antibodies capable of neutralizing PLC activity. Although unable to identify a PLC-neutralizing region on the Cpa, this procedure has proven useful for rapid polyclonal antiserum production/reagent development for research purposes. Because a multitude of antigen targets, linear or conformational, can be designed and readily incorporated into this system, the use of antibody-guided complexes beyond its original platform can be appreciated.

Interestingly, the results suggest that binding to any continuous epitope of the Cpa is sufficient to block its hemolytic activity, but not to neutralize its PLC activity. As only linear peptides were tested, this suggests neutralization requires binding and blocking of one or more conformation-dependent regions on the toxin itself to inhibit PLC functions (18). Removal of the enzymatic activities of toxins required modification of the sequence during recombinant design or chemical inactivation of purified toxins in order to render them safe for previous vaccine efforts. These modifications may alter the conformation of the toxin itself, and therefore efforts to make the tested vaccines safer have actually caused them to be less efficacious (19, 20). Results from this study support previously reported data in which altered toxin used as a vaccine target was unable to induce production of neutralizing antibodies or fully protective immune responses. Due to these findings, targeting a single toxin may not be the answer for controlling NE. Preventing overgrowth of *C. perfringens* by preemptive nutritional and biosecurity control procedures to maintain gut health or developing therapeutics capable to blocking overgrowth of the bacterium itself may, at least for the time being, be more effective solutions.

This antibody-guided immunization technique is designed to target chicken CD40, a unique concept for antiserum production instead of the commonly used mouse, rabbit, or goat hosts. The phylogenic divergence between avian and mammalian systems allows this method to be potentially used for antiserum production against commonly conserved mammalian target epitopes that have previously proven non-immunogenic in mammalian hosts. Avian host systems also permit the collection of eggs, which contain the specific antibody of interest within the yolk, decreasing blood collection requirements and associated stress on the animal. Avian IgG antibodies produced by antibody-guided immunization are suitable for laboratory research use, development of diagnostic assays, and epitope mapping using linear epitopes. Biotinylated peptides can be easily and inexpensively synthesized in as little as a week, incorporated into the antibody-guided complex, and birds can be immunized for an initial serum collection as early as 1 week postimmunization. Conformational epitopes can also be targeted with this system, but would require more time and expense to generate before proceeding to *in vivo* immunizations. Although antiserum production with this method is a viable option with conformational epitopes, it does not lend itself well for epitope mapping. This specific study has provided proof of principle for the use of antibody-guided immunogen complexes to quickly produce antibodies for epitope mapping verification.

## Ethics Statement

This study was carried out in accordance with the recommendations of animal procedure permit 2013-0254 of Texas A&M University’s Institutional Animal Care and Use Committee (IACUC).

## Author Contributions

CV contributed to the design, laboratory assays, data analysis, and drafted the manuscript. W-KC contributed to preparation of the immunization complexes. VK contributed to the live animal handling and sample collection. BH, LBielke, and LBerghman contributed to the experimental design, peptide design, provided funding and facilities, and evaluating data results.

## Disclaimer

The funder was not involved in the study design or collection, analysis, or interpretation of the data.

## Conflict of Interest Statement

The research described in this manuscript was financially supported by Pacific GeneTech, Ltd. with headquarters in Hong Kong. A PCT application with International Patent Publication No. WO2015/187969 has been filed. The reviewer, YS, and handling editor declared their shared affiliation and the handling editor states that the process nevertheless met the standards of a fair and objective review.
